# A novel, simplified protected Ross technique: The “Seattle shawl” procedure

**DOI:** 10.1016/j.xjse.2024.100014

**Published:** 2024-07-14

**Authors:** Christopher R. Burke, David Mauchley, Scott DeRoo

**Affiliations:** a Division of Cardiothoracic Surgery, University of Washington, Seattle, Wash; b Division of Cardiac Surgery, Seattle Children's Hospital, Seattle, Wash

**Keywords:** aortic valve, Ross procedure, surgical technique

## Abstract

**Objective:**

Autograft dilation and subsequent aortic regurgitation remain a challenge in the long term after the Ross procedure in adults. We have developed a novel technique for a modified Dacron wrap of the pulmonary autograft to help stabilize and mitigate this issue.

**Methods:**

In 2022, we formalized a patient-specific tailored algorithm for autograft protection during the Ross procedure, including use of a modified Dacron wrap (“Seattle shawl”). This technique involves the use of a Valsalva graft, which is “keyholed” to facilitate coronary button implantation on the autograft and then wrapped around the pulmonary autograft. A total of 6 patients have undergone the Seattle shawl procedure. Mean follow-up time was 11.8 months (range, 4-17 months).

**Results:**

All 6 patients had bicuspid aortic valves with severe aortic regurgitation. Mean aortic annulus size was 30.3 mm, and mean aortic root diameter was 4.4 cm. There were no perioperative strokes, reinterventions, or mortalities. All patients had mild or less aortic regurgitation on the most recent echocardiogram. Aortic root diameters were between 3.2 and 3.9 cm on the most recent imaging examination. There were no mortalities or complications observed during the follow-up period.

**Conclusions:**

Pulmonary autograft dilation remains a challenge postoperatively after the Ross procedure in adults. Multiple interventions have been used to minimize this phenomenon. These include technical modifications such as annuloplasty rings or complete inclusion of the autograft within a Dacron graft. We present a novel, straightforward technique for autograft protection using a modified Valsalva graft. Early outcomes in 6 patients appear promising with this technique.

**Figure:**
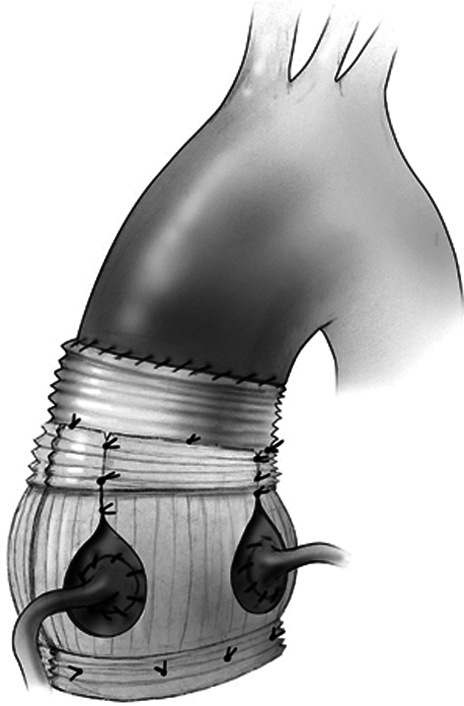
Seattle shawl modified Dacron wrap of pulmonary autograft during Ross procedure.

**Central Message**

We have a devised a simplified, novel method of a modified Dacron wrap of a pulmonary autograft during the Ross procedure to mitigate future autograft dilation.

**Perspective**

Pulmonary autograft dilation remains a source of long-term Ross failure in adults. Several technical modifications have been devised to combat this issue. We have developed a patient-tailored approach to autograft protection, including a novel method for a simplified Dacron wrap using a Valsalva graft. Early outcomes are promising, and we hypothesize this will decrease rates of future autograft dilation.

Recent reports of excellent medium- and long-term outcomes for the Ross procedure in young and middle-aged patients have led to a resurgence of interest in this operation.^1^^,^^2^ The Ross procedure remains the only aortic valve substitute that can restore normal life expectancy in this patient population.^3^ However, concerns remain over both the complexity of this operation (and thus widespread generalizability) and the durability.

The Ross procedure is a technically demanding operation that has been shown to have excess perioperative mortality when performed at low-volume centers.^4^ Further, pulmonary autograft dilation in the long term has been a well-recognized phenomenon and a source of late autograft failure.^5^^,^^6^ This has led some surgeons to modify the Ross technique in adults, by adding aortic annuloplasty rings and in some cases performing an autograft inclusion within a Dacron graft.^7^, ^8^, ^9^, ^10^ Certain patient populations have been identified as high risk for late autograft dilation, including those with bicuspid aortic valve (BAV), aortic regurgitation (AR) predominant disease, and a dilated aortic annulus.^11^ However, addition of autograft inclusion within a Dacron graft is not without potential problems, as some have cited concerns regarding coronary implantation with this technique.^10^

We have developed an individualized, patient-specific approach to pulmonary autograft protection during the Ross procedure (Figure 1). In select patients deemed higher risk for late autograft dilation, we have developed a simplified technique for “partial wrapping” of the autograft using a modified Valsalva graft (Terumo Medical Corp) with keyhole incisions to facilitate the coronary buttons. This straightforward modification adds little to the freestanding autograft implant technique and stabilizes the autograft at the annular, neosinus, and neo–sinotubular junction (STJ) level. We have used this novel technique on 6 patients with promising early results.

**Figure 1:**
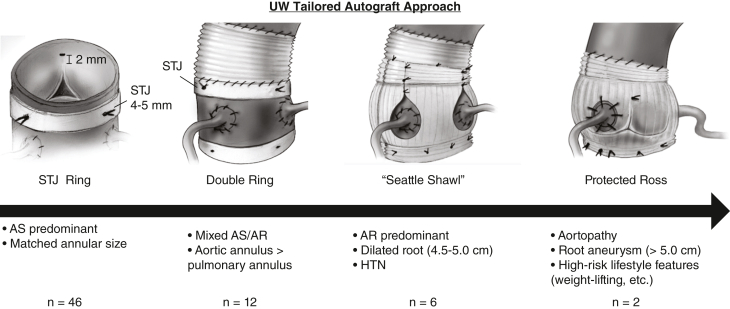
Patient-tailored approach to autograft protection during the Ross procedure in adults. *AR*, Aortic regurgitation. *STJ*, sinotubular junction; *AS*, aortic stenosis; *HTN*, hypertension.

## Material and Methods

In April 2022, we instituted a patient-specific approach to pulmonary autograft protection for adult Ross procedures (Figure 1). Several clinical and anatomic factors are considered when planning the surgical approach. In patients deemed higher (but not highest) risk for future autograft dilation, we developed a novel modified Dacron wrap of the pulmonary autograft to provide an efficient, simplified method of autograft stabilization (Seattle shawl). To date, we have used this technique in 6 adult patients who underwent the Ross procedure. Clinical, operative, and outcomes data were reviewed. To compare operative times, a cohort of “freestanding autograft” implantation in patients who underwent the Ross was generated by including such patients operated on between the first and last patients who received the Seattle shawl. This retrospective cohort study was approved by the University of Washington Institutional Review Board (STUDY00014540, 12/6/21). Informed consent requirement was waived.

Our standard surgical approach for a Ross procedure includes central aortic cannulation in the mid arch and 2-stage venous cannulation in the right atrium. We use a low threshold to replace the ascending aorta (typically if > 4.0 cm). We first inspect the pulmonary valve before aortic root dissection if concerns are present on preoperative imaging for pulmonary valve abnormalities. The aortic root is dissected free, and coronary buttons are mobilized. The distal main pulmonary artery is then transected, and the autograft is mobilized in a standard fashion.

**Figure 2:**
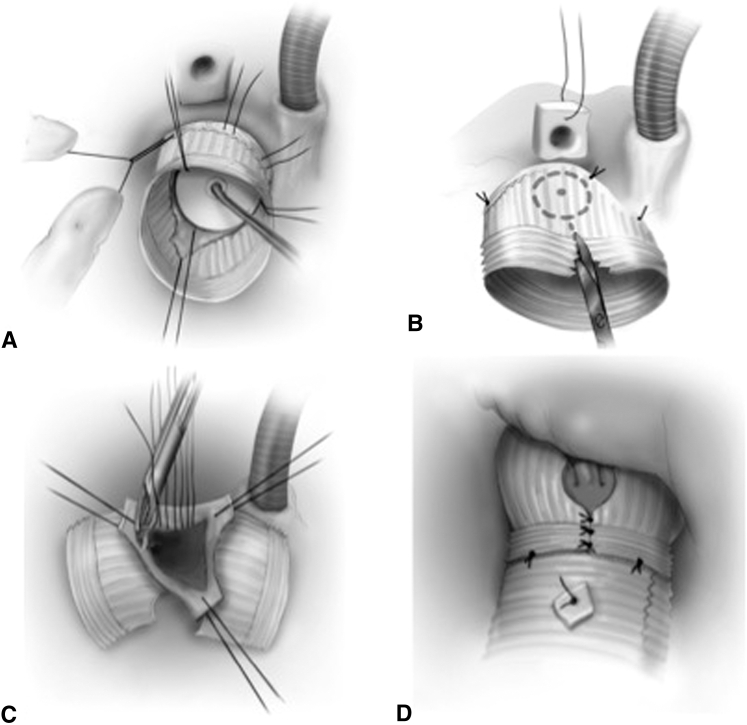
The Seattle shawl technique. A, The Valsalva graft is tied down over a dilator. Dilator size is chosen based on autograft annulus size and final desired “aortic annulus” size. B, Keyhole incisions are made to accommodate the left and right coronary buttons. C, A standard, freestanding autograft implantation of the pulmonary autograft is performed. Our preferred method involves interrupted sutures and routine placement of a Dacron STJ ring. D, At the conclusion of the case, after hemostasis is achieved, the “flaps” of the Valsalva shawl are tacked to the STJ ring, native ascending aorta, or ascending aortic graft (if used). *STJ*, Sinotubular junction.

For the Seattle shawl (modified Dacron wrap) procedure, a deep external aortic root dissection to the level of the virtual basal ring is performed. Sizing of the Valsalva graft (and therefore the annuloplasty performed) is critical to match pulmonary autograft geometry. The pulmonary autograft annulus is measured after harvest, and a Valsalva graft is sized by adding 6 to 7 mm from the desired annulus size (typically a 32- or 34-mm graft). The skirt of the Valsalva graft is trimmed to approximately 5 mm. Six mattress sutures are then placed around the annulus at the level of the basal ring and passed through the skirt of the graft. The graft is tied down over a dilator that matches the pulmonary autograft annular size to achieve the goal reductive annuloplasty. The Valsalva graft is then carefully keyholed to later accommodate the left and right coronary buttons. At this point, the flaps that were created in the Valsalva graft are set aside (Figure 2).

**Figure 3:**
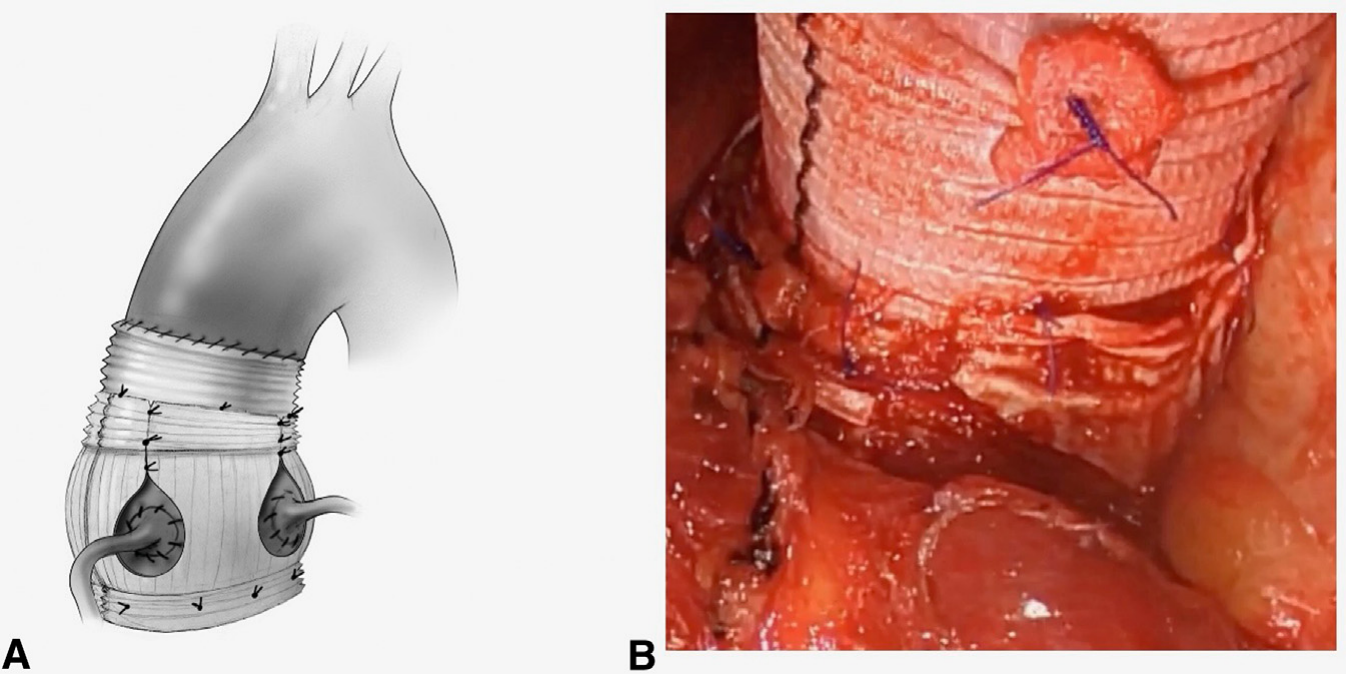
A, Schematic of Seattle shawl technique (A) and intraoperative photograph of Seattle shawl modified Dacron wrap tacked to an ascending aortic graft (B).

The Ross procedure then proceeds as a typical freestanding autograft implant. We use interrupted sutures and place an STJ Dacron ring on all patients to help reinforce the autograft-aortic suture line and stabilize the STJ in the long term. We began placing an STJ ring on all Ross patients in an effort (1) to help support the autograft-aortic suture line and prevent bleeding and pseudoaneurysm formation, and (2) to help prevent subsequent STJ dilation and recurrent AR. A pulmonary homograft (Synergraft, Artivion Inc) is used to reconstruct the pulmonary valve. The patient is then separated from bypass, and heparin is reversed. Once hemostasis is confirmed, the 2 flaps of the Valsalva graft are brought together and carefully tacked to the STJ ring or ascending aorta/ascending graft (if used) (Figure 3). We make a point to get as near circumferential as possible regarding the wrap. The Video 1 shows a full description of our operative technique.

Postoperatively, patients are maintained on strict blood pressure control beginning immediately after the crossclamp is removed. In general, patients aged less than 50 years are maintained at a systolic blood pressure (SBP) less than 110 mm Hg, and those aged more than 50 years are given a goal SBP less than 120 mm Hg. Consideration for preoperative hypertension and risk of developing acute kidney injury is given, especially in an “older” Ross population. Intravenous nicardipine is started as needed in the intensive care unit. First-line oral agents include labetalol and captopril, followed by hydralazine as a third-line agent. Strict blood pressure control is maintained for 6 to 12 months depending on the patient's clinical condition.

## Results

This technique has now been completed on 6 patients. All were male, and all had a BAV and presented with severe AR. Patient demographics are shown in Table 1. One patient (#5) had previous cardiac surgery: a BAV repair in adolescence. Anatomic and operative characteristics are shown in Table 2. Aortic annulus size ranged from 29 to 32 mm. Aortic root diameters ranged from 4.1 to 4.7 cm (Table 2). A comparison of operative times with a “freestanding autograft” implantation cohort revealed an aortic crossclamp time of 189.5 minutes in the Shawl group versus 175 minutes in the freestanding autograft group (Table 3).

**Table 1:** Patient demographics

Patient	Age, y	Sex	Weight (kg)	Height (cm)	BSA	HTN	HL	COPD	CKD	Arrythmia/atrial fibrillation	Tobacco	EtOH	Illicit drugs	Active endocarditis	Previous cardiac surgery
1	45	M	87	193	2.2	N	N	N	N	N	N	N	N	N	N
2	35	M	97	173	2.1	N	N	N	N	Y	N	N	N	N	N
3	23	M	67	175	1.8	N	N	N	N	N	N	N	N	N	N
4	32	M	105	183	2.3	Y	N	N	N	N	N	N	N	N	N
5	39	M	85	184	2.1	N	N	N	N	N	N	N	N	N	Y
6	35	M	105	191	2.4	Y	N	N	N	Y	N	N	Y	N	N
Median	**35**		92	183.5	2.15										

*BSA*, Body surface area; *HTN*, hypertension; *HL*, hyperlipidemia; *COPD*, chronic obstructive pulmonary disease; *CKD*, chronic kidney disease; *EtOH*, alcohol use.

**Table 2:** Anatomic and operative characteristics

Patient	Degree of preoperative AS	Degree of preoperative AR	Ejection fraction (%)	Aortic root size (cm)	Aortic annulus size (mm)	Pulmonary annulus size (mm)	Aortic operation	Size of Valsalva graft (mm)	CPB time (min)	Crossclamp time (min)
1	None	Severe	60	4.4	30	27	Ascending	34	232	176
2	None	Severe	65	4.5	29	27	Ascending	34	222	181
3	Mild	Severe	52	4.1	33	28	Hemiarch	34	234	212
4	None	Severe	55	4.2	30	27	None	34	205	178
5	Severe	Severe	57	4.7	29	25	Ascending	32	236	211
6	Mild	Severe	60	4.4	31	26	Ascending	32	222	198
Median			58.5	4.4	30	27			227	189.5

*AS*, Aortic stenosis; *AR*, aortic regurgitation; *CPB*, cardiopulmonary bypass.

**Table 3:** Comparison of cardiopulmonary bypass and crossclamp times between Shawl technique and freestanding autograft technique

Technique	CPB time(min, median)	Crossclamp time (min, median)
Shawl (n = 6)	227	189.5
Freestanding autograft (n = 27)	206	175

*CPB*, Cardiopulmonary bypass.

**Figure 4:**
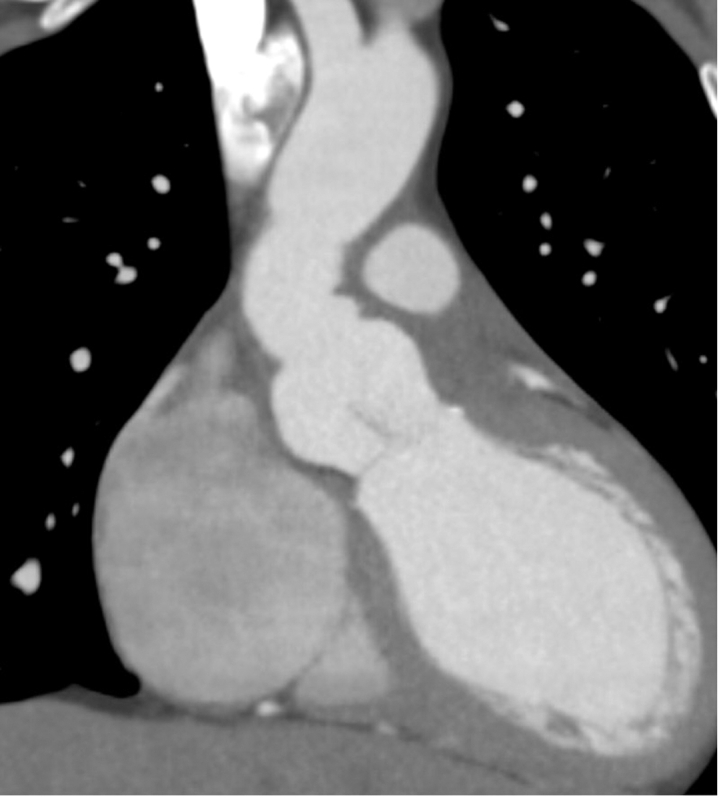
Postoperative computed tomography angiography of autograft 1 year after the Ross with the Seattle shawl technique. The autograft measures 3.7 cm in diameter.

There were no perioperative stroke or deaths. No patient had a significant postoperative adverse event. All were discharged home in good condition, and all continue to do well in the outpatient setting. Follow-up data are shown in Table 4. Mean follow-up time is 11.8 months (range, 4-17 months). The degree of AR at the most recent echocardiogram ranged from none (2 patients), trace (2 patients), to mild (2 patients). No patient has required any reintervention to date. All aortic root dimensions measured less than 4.0 cm on the most recent echocardiogram (Table 4 and Figure 4).

**Table 4:** Postoperative echocardiogram outcomes

Patient	Postoperative AR	Postoperative peak velocity (m/s)	Postoperative peak LVOT velocity (m/s)	Postoperative aortic root size (cm)	Postoperative AR
1	Trace	1.4	1.1	3.7	Trace
2	Mild	1.4	1.1	3.7	Mild
3	Mild	1	0.8	3.2	Mild
4	Trace	1.2	0.9	3.9	Trace
5	None	1.4	NA	3.5	None
6	None	1.2	0.7	3.3	None
Median		1.3	0.9	3.6	

*AR*, Aortic regurgitation; *LVOT*, left ventricular outflow tract.

## Discussion

Renewed interest in the Ross procedure in adults has led to a significant increase in the application of this procedure during the past 5 years. Although it remains a technically demanding operation, excellent results have been observed at high-volume centers. However, there are many lessons that should be learned from previous iterations of this operation. One of the major long-term drawbacks that has been observed in patients post-Ross is autograft dilation with subsequent AR.^5^^,^^6^^,^^12^

Surgeons have devised several methods to combat autograft dilation.^13^^,^^14^ In the freestanding autograft implantation technique, modifications include a deep left ventricular outflow tract implant, trimming of all excess pulmonary artery tissue, and strict postoperative blood pressure control (often SBP < 110 mm Hg for up to 1 year). Others have devised various ring and suture annuloplasty techniques, whereas some groups have advocated for autologous external autograft stabilization.^15^ Still others have described full Dacron inclusion techniques with the autograft sutured within a graft.^9^^,^^16^ All of these modifications have shown promising results but are not without potential issues. These include technical complexity, increased difficulty at reoperation, and potential for coronary button issues. Carrel and Kadner^17^ reported 9-year follow-up after protected Ross using Dacron inclusion and found a 95% freedom from autograft reintervention rate and 68% of patients had trace/zero AR at last follow-up. No patients had subsequent autograft dilation.^17^

We have devised an individualized, patient-tailored autograft approach for Ross procedures in adults. Only the highest risk patients (genetic aortopathy, root aneurysm, other high-risk features) receive a full Dacron jacket with autograft inclusion within a graft. We perform full Dacron inclusion technique in these highest risk patients given both the aortic and pulmonary roots are derived from the embryologic truncus arteriosus; therefore, we believe these patients’ autografts would be at extremely high risk to dilate over time with any freestanding root implantation technique. We believe there are some downsides to this technique, including increased operative time and technical complexity, especially with handling the coronary buttons. Also, creation of a potential space within the neoaortic root can create long-term problems with autograft distortion or coronary artery kinking should fluid collect in this space. We think the Seattle shawl technique obviates many of these concerns by allowing straightforward coronary button anastomoses and no contained potential space between the autograft and the Dacron graft is created.

We devised a simplified technique for Dacron autograft support in patients undergoing the Ross who are deemed high (but not highest) risk for future autograft dilation. This involves annuloplasty using the skirt of a Valsalva graft followed by keyholing the graft to allow straightforward coronary button implantation. This concept is similar to the Florida sleeve technique, which has shown promising long-term results in patients with aortic root aneurysm.^18^, ^19^, ^20^ We believe the Seattle shawl technique to be a reproducible and straightforward adjunct to a freestanding autograft implantation during a Ross procedure. Early outcomes in 6 patients suggest good autograft valve function and stable neoaortic root dimensions in the first 2 years of follow-up.

### Study Limitations

Several limitations deserve mention. This is a small retrospective study on a novel surgical technique. Mean follow-up is only 1 year. Longer-term data are needed to understand the true effectiveness of this approach. Further, establishment of procedural “controls” with full Dacron inclusion or freestanding autograft technique in a similar patient population will help establish the true efficacy of the Seattle shawl technique.

## Conclusions

Autograft dilation after the Ross procedure remains a challenge and requires a thoughtful approach from the Ross team. Surgeons have devised several modifications to the surgical technique to address this issue, including annuloplasty rings and Dacron wraps. Further, strict blood pressure control in the postoperative period has been recognized as beneficial to prevent dilation. We have developed a simplified technique to accomplish both ring annuloplasty and a modified Dacron wrap of the autograft using a modified Valsalva graft. This technique has the potential to help stabilize the autograft while adding minimal time and complexity to the procedure. Further prospective study is needed to understand the long-term autograft results with this technique.

## Conflict of Interest Statement

The authors reported no conflicts of interest.

The *Journal* policy requires editors and reviewers to disclose conflicts of interest and to decline handling or reviewing manuscripts for which they may have a conflict of interest. The editors and reviewers of this article have no conflicts of interest.

## Supplementary Data

Video 1 Procedural video of Ross procedure with "Seattle shawl" modified Dacron wrap. Video available at: https://www.jtcvs.org/article/S2950-6050(24)00014-7/fulltext.
