# Supplementary figures and images for: A novel, simplified protected Ross technique: The “Seattle shawl” procedure
Source: JTCVS Struct Endovasc. 2024 Jul 14;1-2:100014. doi: 10.1016/j.xjse.2024.100014 (PMC13244721; doi:10.1016/j.xjse.2024.100014)

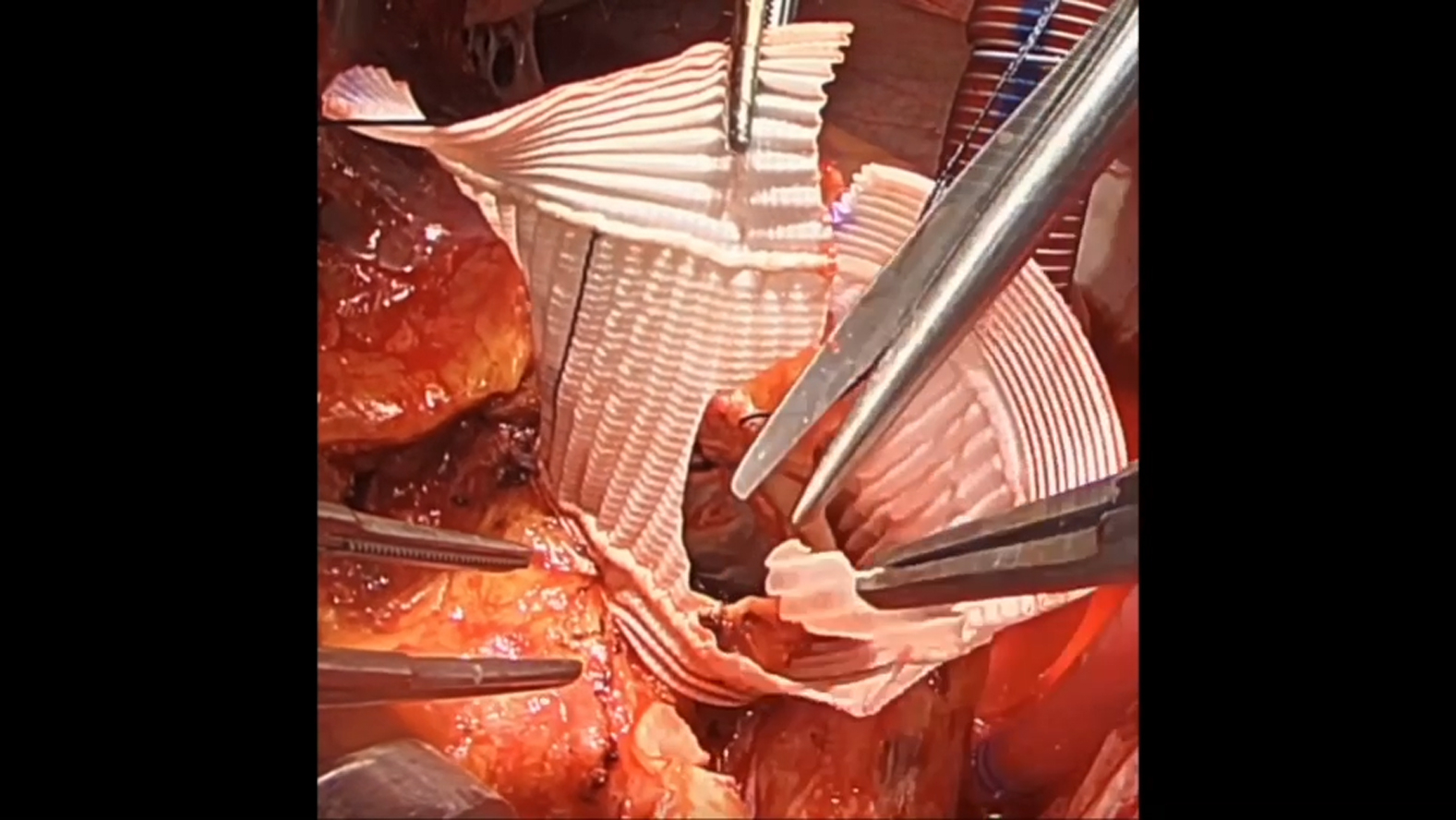

Supplement: Video 1 — Procedural video of Ross procedure with "Seattle shawl" modified Dacron wrap. Video available at: https://www.jtcvs.org/article/S2950-6050(24)00014-7/fulltext. [file fx2.jpg]
